# Comparative Long-Term Outcomes of Vertebroplasty Versus Kyphoplasty for Osteoporotic Vertebral Compression Fractures: A Propensity-Matched Analysis

**DOI:** 10.7759/cureus.96438

**Published:** 2025-11-09

**Authors:** Nicholas G Belt, Austin Lee, Parshva Sanghvi, R. Justin Mistovich, Jonathan Belding

**Affiliations:** 1 Orthopedic Surgery, Case Western Reserve University School of Medicine, Cleveland, USA; 2 Orthopedic Surgery, MetroHealth Medical Center, Cleveland, USA

**Keywords:** cement leakage, kyphoplasty, osteoporotic vertebral compression fracture (ovcf), postoperative outcomes, vertebroplasty

## Abstract

Introduction

Vertebroplasty and kyphoplasty are widely performed for osteoporotic vertebral compression fractures (VCFs). The objective of this study was to compare long-term clinical outcomes between patients undergoing vertebroplasty and kyphoplasty for osteoporotic VCFs.

Methods

We used the TriNetX US Collaborative Research Platform to identify adults aged ≥50 years with osteoporotic VCFs treated with vertebroplasty or kyphoplasty between 2000 and 2025. Patients with malignancy were excluded. Cohorts were propensity-matched 1:1 (7,528 per cohort) on demographic, comorbidity, and bone health variables. Outcomes were assessed using International Classification of Diseases-10 (ICD-10) and Current Procedural Terminology (CPT) codes: subsequent fractures (one year), short-term neurologic/functional complications (one month), opioid prescriptions (one year), repeat augmentation (two years), and all-cause mortality (five years).

Results

Patients treated with kyphoplasty had a higher one-year risk of subsequent vertebral fractures compared with vertebroplasty (4,196 (55.7%) vs 3,905 (51.9%); HR 0.89; 95% CI 0.85-0.93; p < 0.001). Patients undergoing vertebroplasty experienced more short-term neurologic complications, including spinal cord compression (HR 1.19; 95% CI 1.08-1.31; p < 0.001), radiculopathy (HR 1.19; 95% CI 1.06-1.33; p = 0.004), and difficulty walking (HR 2.15; 95% CI 1.65-2.79; p < 0.001). The two-year risk of repeat vertebral augmentation did not differ meaningfully between procedures (1,111 (14.8%) vs 1,069 (14.2%); HR 1.00; 95% CI 0.90-1.10; p = 0.90). Five-year all-cause mortality was similar between vertebroplasty and kyphoplasty (HR 1.04; 95% CI 0.99-1.10; p = 0.13).

Conclusion

Vertebroplasty and kyphoplasty demonstrated comparable long-term survival and repeat procedure rates, but differed in specific risk profiles. Kyphoplasty carried a modestly higher risk of subsequent fractures, whereas vertebroplasty was associated with more short-term complications and earlier, more frequent opioid use. These findings should inform procedure selection in patients with osteoporotic VCFs.

## Introduction

Background

Vertebral compression fractures (VCFs) affect an estimated 1.5 million adults annually in the United States [1]. About one-quarter of postmenopausal women will sustain a VCF in their lifetime, and prevalence approaches 40% by age 80 [2,3], with most fractures attributable to osteoporosis [4]. VCFs can cause severe back pain, functional impairment, and progressive thoracolumbar kyphosis, leading to secondary issues, including poor nutrition, reduced pulmonary capacity, sedentary behavior, and, in some cases, spinal cord compression with neurologic deficits [5,6]. Although nonoperative care is first-line, persistent pain and disability often prompt surgery. Two minimally invasive augmentation procedures - vertebroplasty and balloon kyphoplasty - have been used for decades. The safety and efficacy of these procedures have been both supported and questioned by multiple systematic reviews, clinical trials, cost-effectiveness studies, and quality-of-life studies published in the literature [1,2,6-11]. However, it is important to note that the majority of existing studies compare vertebroplasty or kyphoplasty with nonoperative medical management. There have been only a few prospective RCTs comparing the safety and efficacy of vertebroplasty with kyphoplasty, with limitations including small enrollment and underpowered trial design [8]. Therefore, although there are existing systematic reviews and meta-analyses directly comparing vertebroplasty and kyphoplasty, these data are subject to major limitations due to heterogeneity of results, which reflects the largely nonrandomized patient cohorts included [4,8].

Rationale

To address these limitations, we conducted a retrospective, propensity score-matched cohort study using a large national electronic health record (EHR) database to compare long-term outcomes between vertebroplasty and kyphoplasty for osteoporotic VCFs. We asked whether there are differences in subsequent thoracic or lumbar fractures, short-term neurologic complications and related decompression procedures, repeat vertebral augmentation, and long-term all-cause mortality.

## Materials and methods

Study design

We performed a retrospective cohort study using the TriNetX US Collaborative Research Network, a federated, de-identified EHR platform that aggregates data from more than 60 healthcare organizations across the United States. The study period spanned January 1, 2000, through May 1, 2025. Because the data were aggregated and de-identified, this study was deemed exempt from review by the Western Institutional Review Board (IRB) by a qualified expert, as defined in Section §164.514(b)(1) of the HIPAA Privacy Rule. 

Participants and study subjects

Patient cohorts were created on April 12, 2025, using Current Procedural Terminology (CPT) codes to identify adult patients who underwent thoracic or lumbar vertebroplasty or kyphoplasty. Vertebroplasty was defined by CPT codes 22510, 22511, and 22512, while kyphoplasty was defined by CPT codes 22513, 22514, and 22515. To isolate patients treated for osteoporotic VCFs, we excluded individuals under the age of 50 and those with a diagnosis of multiple myeloma (C90), primary malignant neoplasm of bone and articular cartilage (C40-C41), or secondary malignant neoplasm of bone or bone marrow (C79.5). The final cohorts included only patients aged 50 and older who underwent vertebral augmentation for nonmalignant fractures.

Description of experiment

Patients underwent either vertebroplasty or balloon kyphoplasty for osteoporotic VCFs, as identified using CPT codes within the TriNetX US Collaborative Research Network. Both procedures are percutaneous vertebral augmentation techniques, in which polymethylmethacrylate cement is injected into the fractured vertebral body. Kyphoplasty uses an inflatable balloon tamp to create a cavity, whereas vertebroplasty involves direct cement injection under fluoroscopic guidance. Because this was a retrospective database study, no standardized surgical technique, cement volume, or cement type could be controlled, and outcomes reflect real-world practice across participating health systems.

Variables and outcome measures

Outcomes were defined using International Classification of Diseases-10 (ICD-10) and CPT codes, as detailed in Table 1. The primary outcomes included subsequent thoracic or lumbar vertebral fractures, repeat vertebral augmentation procedures, spinal impingement syndromes (such as radiculopathy or spinal cord compression), decompressive spinal surgery (laminectomy), opioid prescription frequency, and all-cause mortality. Subsequent vertebral fractures were assessed up to one year following the index procedure. Repeat vertebral augmentation procedures were evaluated within two years of the initial intervention. Spinal impingement diagnoses and decompressive spinal surgeries were assessed within one month of the procedure to increase specificity. Opioid prescription frequency was assessed within one year post-procedure, and all-cause mortality was assessed up to five years post-procedure. These timeframes were selected to balance clinical relevance with the ability to capture events likely related to the index procedure.

**Table 1:** ICD-10 and CPT Codes Used to Define Clinical Outcomes ICD-10, International Classification of Diseases-10; CPT, Current Procedural Terminology

Outcomes	ICD-10 or CPT Codes	Description
Back Pain	M54.5	Low back pain
	M54.6	Pain in thoracic spine
Muscle Weakness	M62.81	Muscle weakness
Difficulty Walking	R26.2	Difficulty walking
Subsequent Fractures	S22.0	Fracture of thoracic vertebra
	S32.0	Fracture of lumbar vertebra
Spinal Cord Compression	G95.2	Cord Compression
	G95.29	Other Cord Compression
	M48.04	Spinal Stenosis, thoracic region
	M48.05	Spinal Stenosis, thoracolumbar region
	M48.06	Spinal stenosis, lumbar region
Radiculopathy	M54.14	Radiculopathy, thoracic region
	M54.15	Radiculopathy, thoracolumbar region
	M54.16	Radiculopathy, lumbar region
	M54.17	Radiculopathy, lumbosacral region
Vertebroplasty	22510	Percutaneous vertebroplasty, 1 vertebral body; thoracic
	22511	Percutaneous vertebroplasty, 1 vertebral body; lumbar
	22512	Percutaneous vertebroplasty, 1 vertebral body; each additional vertebral body
Kyphoplasty	22513	Kyphoplasty, 1 vertebral body; thoracic
	22514	Kyphoplasty, 1 vertebral body; lumbar
	22515	Kyphoplasty, 1 vertebral body; each additional vertebral body
Laminectomy	63046	Laminectomy, facetectomy and foraminotomy, single vertebral segment; thoracic
	63047	Laminectomy, facetectomy and foraminotomy, single vertebral segment; lumbar
Opioid Prescriptions	RxNorm Code	
	10689	Tramadol
	7804	Oxycodone
	5489	Hydrocodone
	7052	Morphine
	3423	Hydromorphone
	1819	Buprenorphine

Demographics and description of study population

After matching, the mean age at the time of the procedure was 75 years for both groups. Females accounted for 71% of the vertebroplasty cohort and 72% of the kyphoplasty cohort, while males represented 27% of both. The average BMI was 26.8 in both cohorts. The most prevalent comorbidity in both groups was essential hypertension (66%), followed by low back pain (58%), fracture of the lumbar vertebra (51%), and osteoporosis, with or without current fracture, in approximately 72% of patients. Table 2 summarizes the full demographic and clinical characteristics of both cohorts, before and after matching.

**Table 2:** Demographic and Clinical Characteristics of Vertebroplasty and Kyphoplasty Cohorts Before and After Propensity Score Matching Values are presented as mean ± standard deviation (SD) or number (percentage). KP, Kyphoplasty; VP, Vertebroplasty; BM, Before Matching; AM, After Matching; SMD, Standardized Mean Difference; NSAIDs, Nonsteroidal Anti-inflammatory Drugs

		KP BM (n = 24,305)	VP BM (n = 7,544)	SMD BM	KP AM (n = 7,528)	VP AM (n = 7,528)	SMD AM
Age (Mean ± SD)							
	Age at Index	74.8 ± 10.4	75.2 ± 10.8	0.030	75.4 ± 10.3	75.2 ± 10.8	0.019
Sex, n (%)							
	Male	6,737 (28%)	2,039 (27%)	0.016	2,024 (27%)	2,038 (27%)	0.004
	Female	16,741 (69%)	5,394 (72%)	0.057	5,384 (72%)	5,379 (71%)	0.001
Race, n(%)							
	White	20,467 (84%)	6,471 (86%)	0.044	6,420 (85%)	6,465 (86%)	0.017
	Black or African-American	674 (3%)	276 (4%)	0.050	280 (4%)	271 (4%)	0.006
	Asian	903 (4%)	233 (3%)	0.034	232 (3%)	233 (3%)	0.001
	American Indian or Alaska Native	71 (0%)	20 (0%)	0.005	18 (0%)	20 (0%)	0.001
	Unknown	2,190 (9%)	544 (7%)	0.054	578 (8%)	539 (7%)	0.012
Body Mass Index (kg/m²)	Mean ± SD	26.9 ± 6.2	26.8 ± 6.3	0.009	26.7 ± 6.2	26.8 ± 6.3	0.002
Diagnoses, n(%)							
	Diabetes Mellitus	5,569 (23%)	1,658 (22%)	0.022	1,615 (21%)	1,656 (22%)	0.013
	Essential (Primary) Hypertension	14,881 (61%)	4,983 (66%)	0.100	4,971 (66%)	4,967 (66%)	0.001
	Pain in Thoracic Spine	4,685 (19%)	1,421 (19%)	0.011	1,414 (19%)	1,421 (19%)	0.002
	Low Back Pain	13,657 (56%)	4,402 (58%)	0.044	4,309 (57%)	4,390 (58%)	0.022
	Fracture of Lumbar Vertebra	13,488 (55%)	3,831 (51%)	0.095	3,826 (51%)	3,831 (51%)	0.001
	Fracture of Thoracic Vertebra	10,580 (44%)	3,001 (40%)	0.076	2,996 (40%)	3,001 (40%)	0.001
	Osteoporosis Without Current Pathological Fracture	8,528 (35%)	2,874 (38%)	0.062	2,923 (39%)	2,865 (38%)	0.016
	Osteoporosis With Current Pathological Fracture	8,426 (35%)	2,556 (34%)	0.017	2,525 (34%)	2,554 (34%)	0.008
	Vitamin D Deficiency	5,119 (21%)	1,745 (23%)	0.050	1,711 (23%)	1,739 (23%)	0.009
	Malnutrition	2,016 (8%)	775 (10%)	0.068	797 (11%)	765 (10%)	0.014
	Hypercalcemia	929 (4%)	317 (4%)	0.019	303 (4%)	316 (4%)	0.009
	Hypocalcemia	768 (3%)	238 (3%)	0.000	247 (3%)	238 (3%)	0.007
	Chronic Kidney Disease	4,482 (18%)	1,491 (20%)	0.033	1,452 (19%)	1,486 (20%)	0.011
Diagnostics, n(%)							
	Dual-energy X-ray absorptiometry (DXA), bone density scan of the spine	5,974 (25%)	1,873 (25%)	0.006	1,839 (24%)	1,868 (25%)	0.009
Prescriptions, n(%)							
	Opioid Analgesics	20,290 (83%)	6,545 (87%)	0.092	6,497 (86%)	6,529 (87%)	0.012
	Non-opioid Analgesics	19,706 (81%)	6,378 (85%)	0.092	6,288 (84%)	6,362 (85%)	0.027
	NSAIDs	5,490 (23%)	1,896 (25%)	0.069	1,836 (24%)	1,892 (25%)	0.017

Study subjects

**Figure 1:**
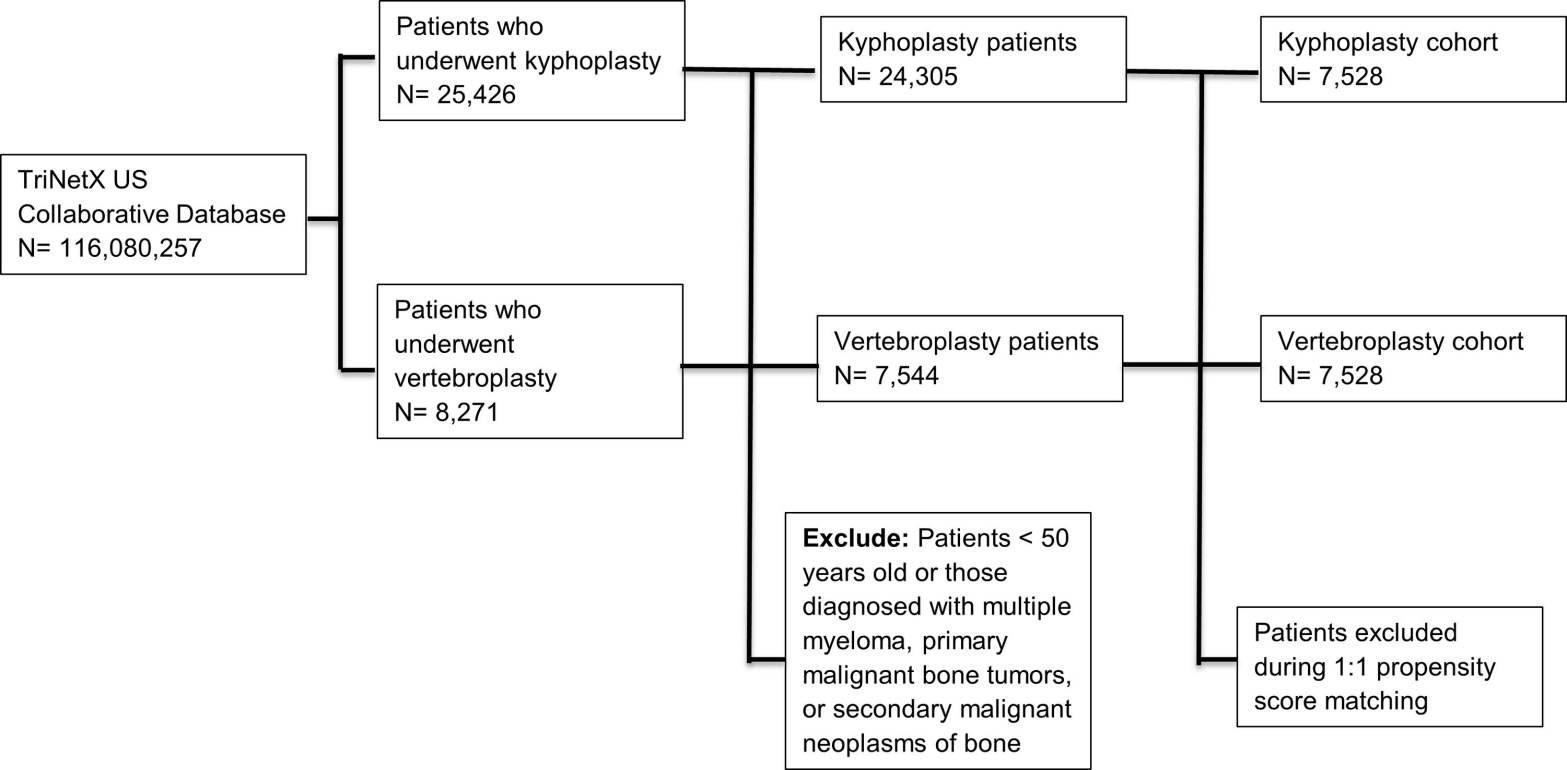
STROBE Diagram The STROBE flow diagram depicts cohort selection from the TriNetX US Collaborative database, the application of inclusion and exclusion criteria, and 1:1 propensity-score matching, yielding the vertebroplasty and kyphoplasty cohorts used in this study.

We initially identified 33,697 patients who underwent vertebral augmentation, including 25,426 who received kyphoplasty and 8,271 who received vertebroplasty. A total of 24,305 patients who underwent kyphoplasty and 7,544 who underwent vertebroplasty were included after applying the inclusion and exclusion criteria (Figure 1). After propensity score matching, 7,528 patients remained in each cohort. The cohorts in this study were matched for several factors, including demographic variables, body mass index (BMI), comorbidities, bone density disorders, spinal diagnoses, vitamin D status, nutritional and electrolyte abnormalities, and use of diagnostic modalities, such as dual-energy X-ray absorptiometry (DXA). A full list of matching variables is available in Table 3, and characteristics before and after matching are summarized in Table 2. These two well-balanced, propensity score-matched cohorts comprised the analytic sample for all outcome analyses.

**Table 3:** ICD-10 and CPT Codes Used for Cohort Matching ICD-10, International Classification of Diseases-10; CPT, Current Procedural Terminology

ICD-10 Code	Description
I10	Essential (primary) hypertension
M54.5	Low back pain
S32.0	Fracture of lumbar vertebra
S22.0	Fracture of thoracic vertebra
M81	Osteoporosis without current pathological fracture
M80	Osteoporosis with current pathological fracture
E55	Vitamin D deficiency
E08-E13	Diabetes mellitus
N18	Chronic kidney disease (CKD)
M54.6	Pain in thoracic spine
E40-E46	Malnutrition
E83.52	Hypercalcemia
E83.51	Hypocalcemia
CPT Code	
1014948	Dual-energy X-ray absorptiometry (DXA), bone density study

Statistical analysis and study size

Propensity score matching (1:1, greedy nearest-neighbor algorithm, caliper width 0.1 SD) was performed to balance baseline demographics, comorbidities, and bone health indicators between cohorts. After matching, 7,528 patients were included in each group. Covariate balance was confirmed using standardized mean differences (<0.1, considered acceptable).

Categorical variables were compared using Chi-square tests, and continuous variables with Mann-Whitney U tests. Time-to-event outcomes were analyzed with Kaplan-Meier methods and Cox proportional hazards regression, with hazard ratios (HRs) and 95% confidence intervals (CIs) reported. All statistical tests were two-tailed, with p < 0.05 considered significant. Analyses were conducted within the TriNetX platform, which uses validated and reproducible statistical pipelines.

## Results

Subsequent thoracic or lumbar fractures (one year)

**Figure 2:**
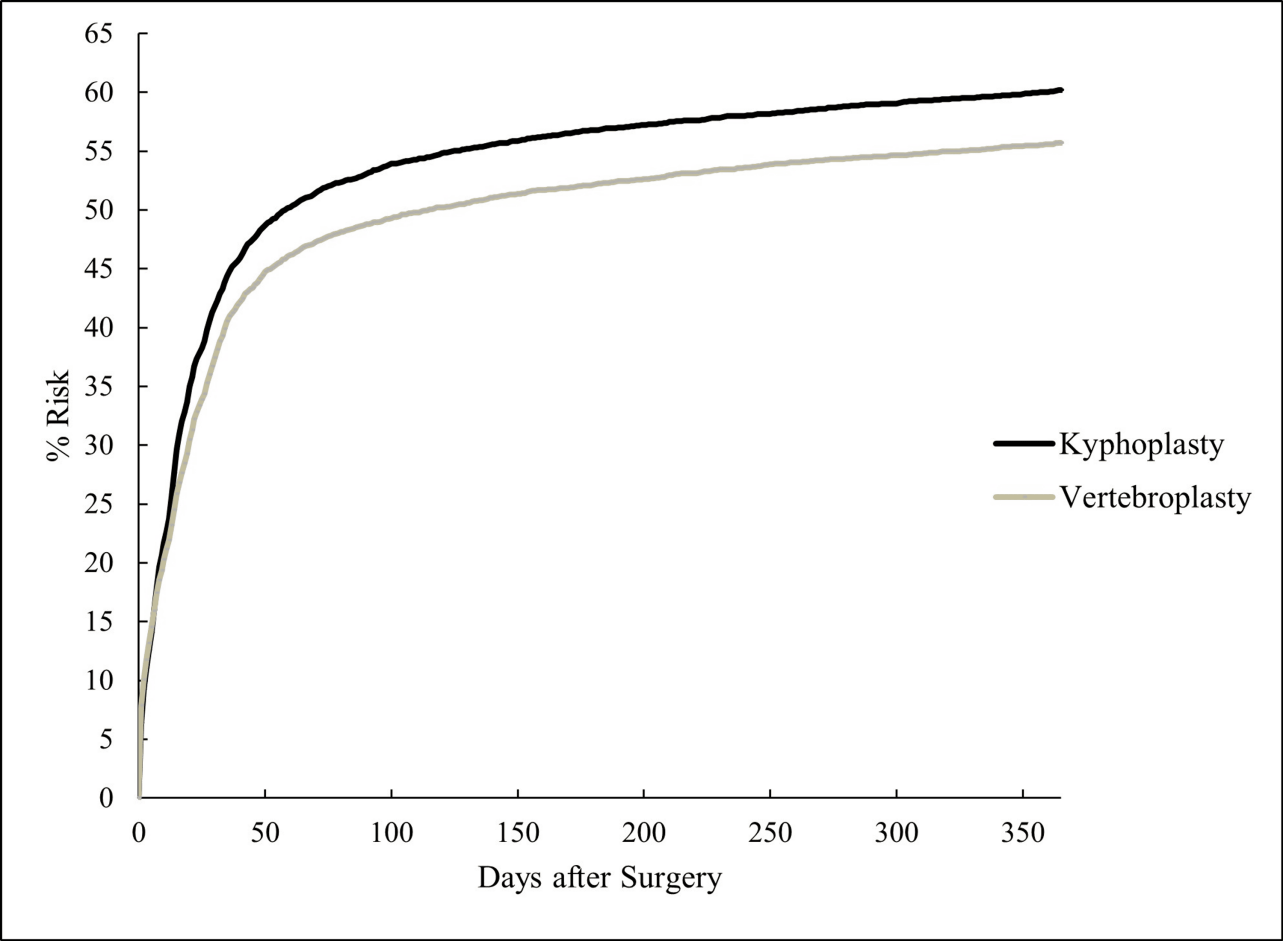
One-Year Risk of Subsequent Thoracic or Lumbar Fractures

Patients who underwent kyphoplasty experienced a higher risk of subsequent thoracic or lumbar fractures within one year compared with vertebroplasty (4,196 (55.7%) vs 3,905 (51.9%); HR 0.89, 95% CI 0.85-0.93; p < 0.001) (Table 4). Interestingly, most patients with subsequent fractures were diagnosed within the two months postprocedure (Figure 2). Specifically, 3,779 (50.2%) of the kyphoplasty cohort and 3,470 (46.1%) of the vertebroplasty cohort were diagnosed with fractures during this period.

**Table 4:** Adverse Outcomes Within One Year Post-augmentation

	Vertebroplasty, n (% Risk)	Kyphoplasty, n (% Risk)	Hazard Ratio, n (95% CI)
Back Pain	3,027 (40.2%)	2,985 (39.7%)	1 (0.9, 1.1)
Fracture of Thoracic or Lumbar Vertebra	3,905 (51.9%)	4,196 (55.7%)	0.89 (0.85, 0.93)

Short-term neurologic or functional complications (one month)

Vertebroplasty was associated with more short-term neurologic complications within one month (Table 5). The risk of spinal cord compression was higher (824 (10.9%) vs 698 (9.3%); HR 1.19, 95% CI 1.08-1.31; p < 0.001), as was radiculopathy (537 (7.1%) vs 452 (6.0%); HR 1.19, 95% CI 1.06-1.33; p = 0.004) and difficulty walking (164 (2.2%) vs 104 (1.4%); HR 2.15, 95% CI 1.65-2.79; p < 0.001). Rates of muscle weakness did not differ between groups. In a subset of patients with spinal cord compression, the severity may have necessitated surgical intervention, as evidenced by laminectomy being performed in 151 (2.0%) of vertebroplasty patients and 135 (1.8%) of kyphoplasty patients.

**Table 5:** Short-Term (One Month) Neurologic and Functional Outcomes Following Vertebral Augmentation

	Vertebroplasty, n (% Risk)	Kyphoplasty, n (% Risk)	Hazard Ratio, n (95% CI)
Radiculopathy	537 (7.1%)	452 (6.0%)	1.19 (1.06, 1.33)
Muscle Weakness	107 (1.4%)	113 (1.5%)	1 (0.75, 1.25)
Difficulty Walking	164 (2.2%)	104 (1.4%)	2.15 (1.65, 2.79)
Spinal Cord Compression	824 (10.9%)	698 (9.3%)	1.19 (1.08, 1.31)

Repeat vertebral augmentation (two years)

**Figure 3:**
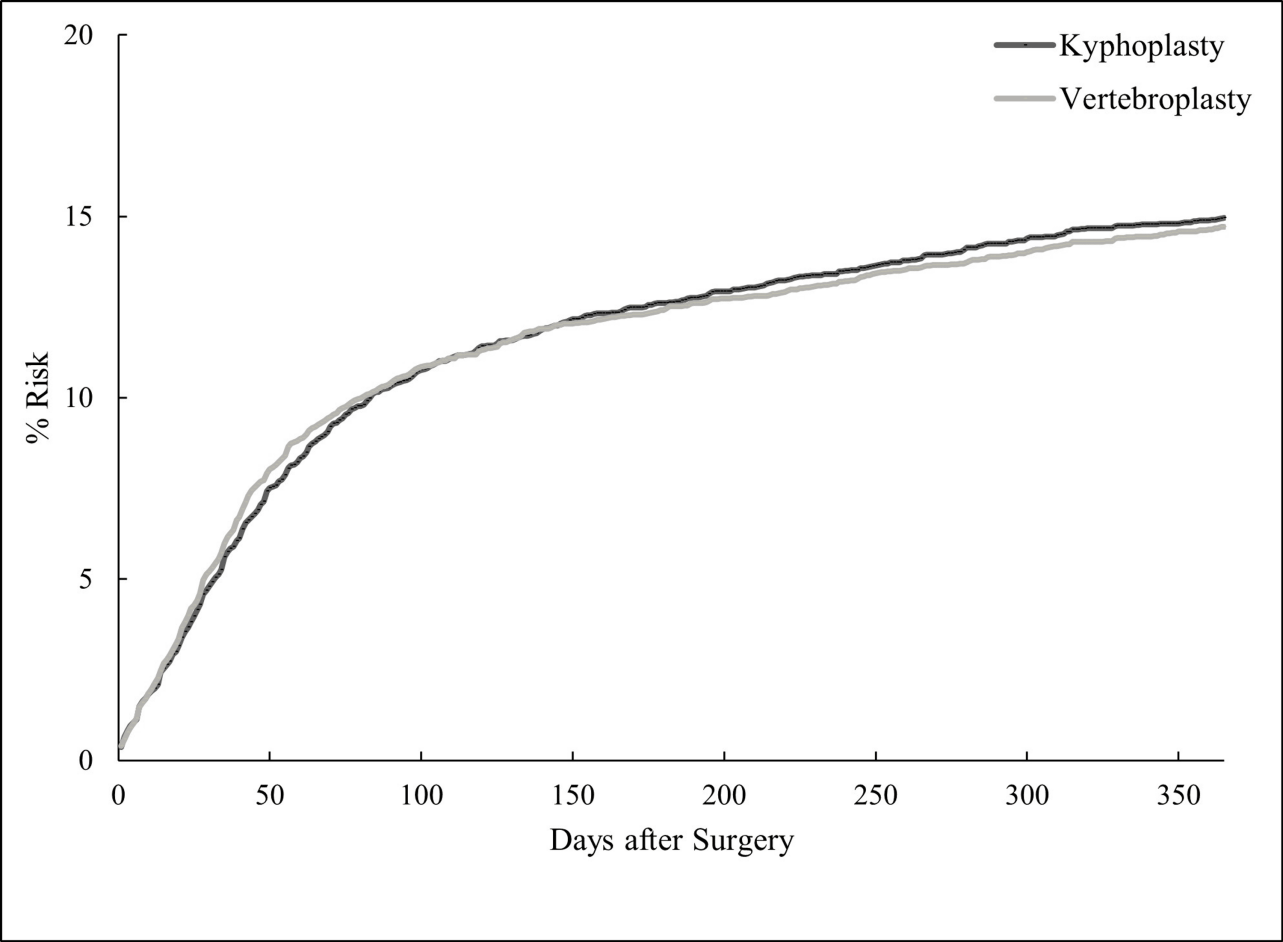
One-Year Risk of Repeat Vertebral Augmentation Procedures After the Index Surgery

The two-year risk of repeat vertebral augmentation was similar between vertebroplasty and kyphoplasty (1,111 (14.8%) vs 1,069 (14.2%); HR 1.00, 95% CI 0.90-1.10; p = 0.90) (Table 6). When repeat procedures occurred, most patients underwent the same procedure as their index surgery. Most repeat procedures occurred within the first three months of the initial procedure (Figure 3), during which approximately 750 (10%) of patients in both cohorts underwent a second vertebral augmentation.

**Table 6:** Risk of Subsequent Vertebral Augmentation Procedures Within Two Years

	Vertebroplasty, n (% Risk)	Kyphoplasty, n (% Risk)	Hazard Ratio, n (95% CI)
Subsequent Vertebroplasty	823 (10.9%)	273 (3.6%)	3.04 (2.66, 3.48)
Subsequent Kyphoplasty	439 (5.8%)	907 (12.0%)	0.45 (0.4, 0.5)
Subsequent Vertebroplasty or Kyphoplasty	1,111 (14.8%)	1,069 (14.2%)	1 (0.9, 1.1)

All-cause mortality (five years)

**Figure 4:**
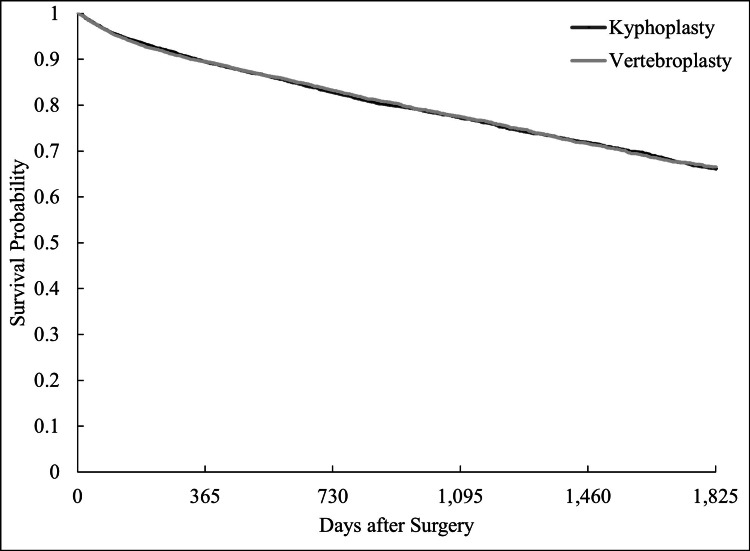
Five-Year Overall Survival After Vertebroplasty and Kyphoplasty

All-cause mortality at five years did not differ between groups (HR 1.04, 95% CI 0.99-1.10, p = 0.13), indicating comparable long-term survival (Figure 4).

Other relevant findings

**Figure 5:**
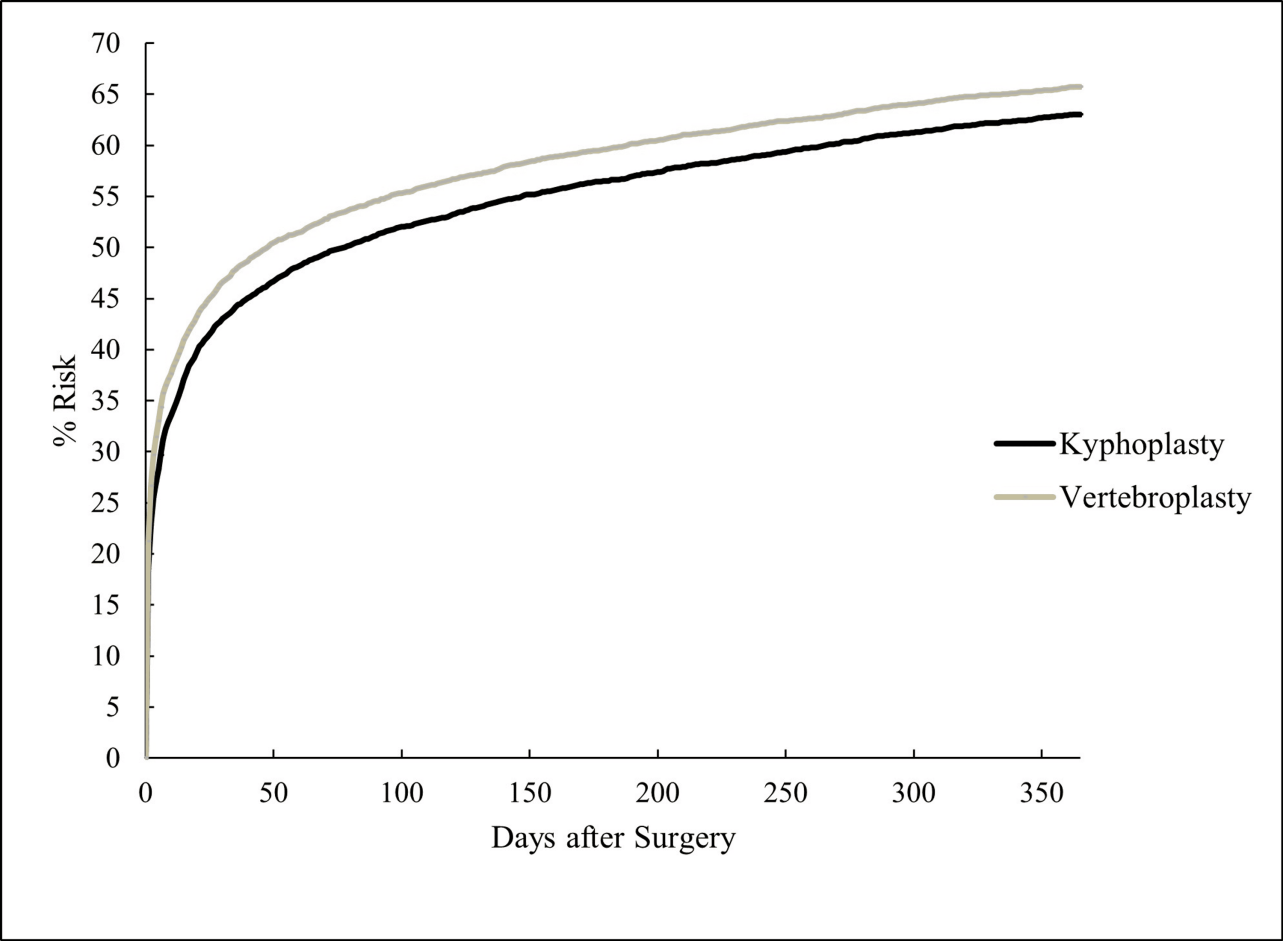
Opioid Prescription Use Within One Year of Index Surgery

Opioid use patterns differed between groups (Figure 5). Patients treated with vertebroplasty were more likely to receive opioid prescriptions within one year (4,667 (62.0%) vs 4,404 (58.5%); HR 1.10, 95% CI 1.05-1.14, p < 0.001), had earlier initiation (median 48 vs 78 days, p < 0.001), and filled more prescriptions overall (mean 8.4 ± 14.9 vs 7.3 ± 13.8, p < 0.001).

## Discussion

Background and rationale

Osteoporotic VCFs are common, debilitating injuries in older adults, and are frequently treated with vertebroplasty or balloon kyphoplasty. Prior studies comparing these procedures have been limited by small sample sizes, heterogeneous designs, and short follow-up. To address these limitations, we conducted a large, retrospective, propensity score-matched cohort study using a national EHR database to compare long-term outcomes of vertebroplasty and kyphoplasty. We found that kyphoplasty was associated with a higher risk of subsequent vertebral fractures, while vertebroplasty was associated with more short-term neurologic complications, and greater postoperative opioid use. Repeat augmentation and long-term all-cause mortality were similar between the procedures.

Limitations

This study had a number of limitations. First, due to the database format, causal relationships between procedures and outcomes cannot be definitively established; however, we attempted to mitigate this issue through carefully selected timeframe criteria, designed to enhance the specificity of the outcomes. Second, there is potential for misclassification due to reliance on coded diagnoses and procedures, as well as the absence of detailed imaging data and clinical variables, such as fracture morphology, vertebral height restoration, and cement volumes. These missing details potentially influence clinical outcomes and procedural success, but are unavailable in our dataset. Third, we were unable to quantify pain intensity or functional disability, evaluate patient-reported outcomes, or specifically identify whether fractures occurred at adjacent levels. These factors limit the completeness of our clinical outcome assessment. Despite these limitations, our study focused on comparing two procedures with well-matched, real-world patient cohorts, which allows meaningful insights into the relative differences between vertebroplasty and kyphoplasty.

Subsequent thoracic or lumbar fractures

The incidence of subsequent thoracic or lumbar vertebral fractures within one year was significantly higher following kyphoplasty (4,196, or 55.7%) compared to vertebroplasty (3,905, or 51.9%). While recurrent fractures are an expected outcome given the natural progression of osteoporosis [7,10-14], the observed modest increase in fracture rates following kyphoplasty may reflect biomechanical alterations resulting from its superior ability to restore vertebral height compared to vertebroplasty [3,6,15]. Specifically, the balloon inflation used in kyphoplasty aims to restore vertebral height, potentially increasing stiffness at the treated vertebra and altering mechanical stresses at adjacent vertebrae [16]. Prior studies have suggested that vertebral height restoration might predispose adjacent vertebrae to increased loading, thus increasing the risk of subsequent fractures [2,16]. Additionally, the observed higher fracture rates post-kyphoplasty may be partially attributable to patients experiencing more rapid symptomatic improvement, allowing earlier participation in physical activities that were previously limited by pain. This abrupt increase in mechanical loads may contribute to new fractures in untreated vertebrae [17]. Patients may also be more prone to falls as they regain mobility, potentially leading to additional vertebral or non-vertebral fractures [14]. However, a recent network meta-analysis found no difference in the risk of adjacent-level fractures between kyphoplasty, vertebroplasty, and natural progression without intervention, suggesting that procedural impact on adjacent fracture risk remains controversial [11].

Short-term neurologic or functional complications

Rates of spinal cord compression and subsequent decompressive surgeries within one month were slightly higher in the vertebroplasty cohort than in the kyphoplasty cohort. Although the absolute difference is small, it is consistent with existing literature reporting higher incidences of cement extravasation associated with vertebroplasty procedures, due to higher injection pressures [8,18,19]. These findings suggest a slightly increased procedural risk of cement extravasation associated with vertebroplasty, although clinically significant events remain rare.

Repeat vertebral augmentation

Interestingly, we observed comparable rates of repeat vertebral augmentation between groups within two years post-procedure. Patients generally underwent the same type of augmentation for subsequent fractures as their initial procedure, potentially reflecting institutional or provider preference. These findings suggest that the long-term durability of vertebroplasty and kyphoplasty is similar and that the need for additional procedures is not influenced by the choice of initial augmentation.

All-cause mortality 

Mortality over a five-year follow-up period was also similar across both cohorts, contrasting with prior observational studies that reported a modest survival benefit associated with kyphoplasty [9,20]. Ultimately, a randomized prospective study directly comparing long-term mortality outcomes between these procedures is needed to clarify this relationship.

Other relevant findings

Opioid prescription patterns within one year following augmentation were different between procedures. Vertebroplasty patients were prescribed opioids sooner postoperatively and had a higher mean number of opioid prescriptions compared with kyphoplasty patients. These differences may indicate variations in early postoperative pain management needs or procedure-specific recovery. Previous randomized trials and meta-analyses comparing these procedures have generally reported no substantial differences in long-term pain relief or functional outcomes [2,8].

## Conclusions

In conclusion, this comparative study demonstrates subtle but clinically relevant differences between vertebroplasty and kyphoplasty in patients with osteoporotic VCFs. Specifically, kyphoplasty was associated with more subsequent thoracic or lumbar fractures at one year, whereas vertebroplasty had higher one-month rates of neurologic or functional complications. Repeat augmentation within two years and five-year all-cause mortality were similar. These findings support shared decision-making that weighs short-term procedural risks against the likelihood of subsequent fractures, while considering patient preferences, bone health optimization, and operator expertise.
